# Supplementary material for: Positive Effects of Organic Amendments on Soil Microbes and Their Functionality in Agro-Ecosystems
Source: Plants (Basel). 2023 Nov 7;12(22):3790. doi: 10.3390/plants12223790 (PMC10674390; doi:10.3390/plants12223790)
Supplement: Supplementary file 1 [file plants-12-03790-s001.zip › plants-2631739-supplementary.pdf]

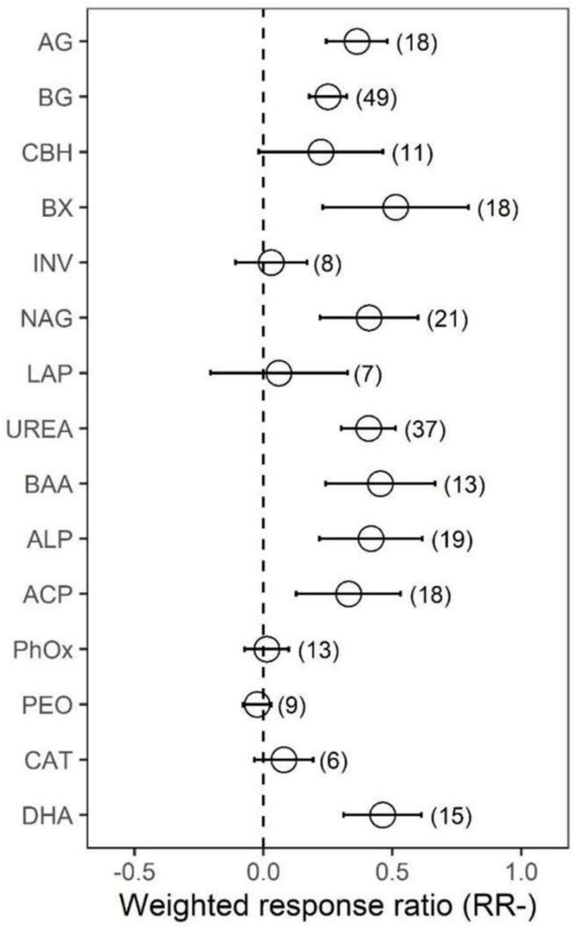

Figure S1. The effect of replacing chemical fertilizer with organic amendments on soil enzyme activities response ratio (natural logarithm-transformed ratio of organic amendments to chemical treatments, RR). The circles with error bars denote the overall mean response ratio and 95% CI, respectively. The number of observations are detailed beside each attribute with parentheses. AG, BG, CBH, BX, INV, NAG, LAP, UREA, BAA, ALP, ACP, PhOx, PEO, CAT and DHA represent α-1,4-glucosidase, β-1,4-glucosidase, β-D-cellobiosidase, β-1,4-xylosidase, invertase, β-1,4 N-Acetyl-glucosaminidase, leucine aminopeptidase, urease, protease, alkaline phosphatase, acid phosphatase, phenol oxidase, peroxidase, catalase and dehydrogenase, respectively.

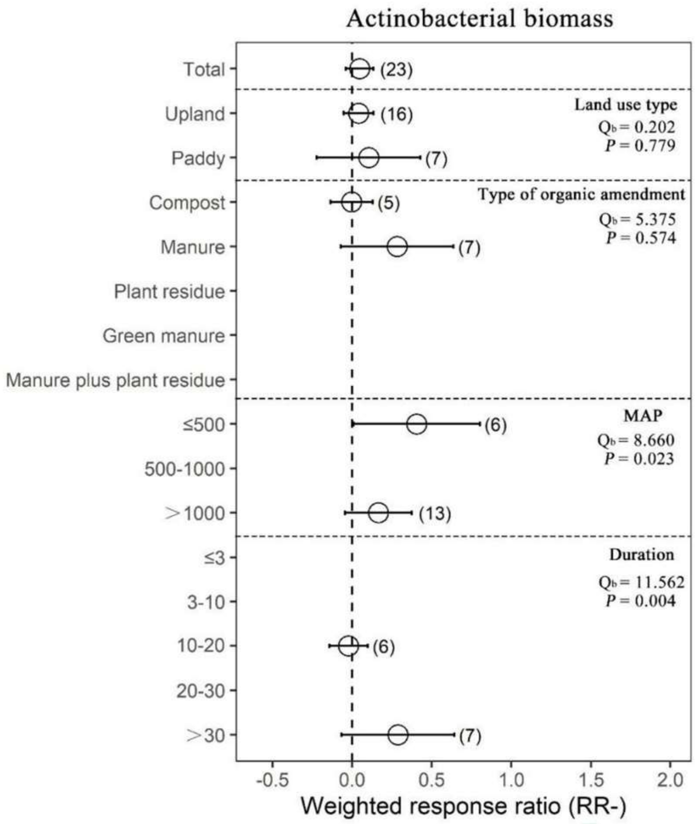

Figure S2. The effect of replacing chemical fertilizer with organic amendments on actinobacterial biomass response ratio (natural logarithm-transformed ratio of organic amendments to chemical treatments, RR). The circles with error bars denote the overall mean response ratio and 95% CI, respectively. The number of observations are detailed beside each attribute with parentheses. The MAP and Duration denotes for mean annual precipitation (mm) and experimental duration (year), respectively. Q_b denotes for between-group heterogeneity in same variable and there is significant difference between groups when $P < 0.05$.
